# Towards deorphanizing G protein-coupled receptors of *Schistosoma mansoni* using the MALAR yeast two-hybrid system

**DOI:** 10.1017/S0031182019001756

**Published:** 2020-07

**Authors:** Oliver Weth, Simone Haeberlein, Martin Haimann, Yinjie Zhang, Christoph G. Grevelding

**Affiliations:** 1 Insitute for Parasitology, Justus Liebig University Giessen, Giessen, Germany; 2 Laboratory of Regeneromics, School of Pharmacy, Shanghai Jiao Tong University, Shanghai, China

**Keywords:** G protein-coupled receptors, neuropeptide, *Schistosoma mansoni*, yeast two-hybrid

## Abstract

Schistosomiasis is an acute and chronic disease caused by parasitic worms of the genus *Schistosoma*. Treatment is solely dependent on praziquantel. In the face of the worldwide dimension, projects have been initiated to develop new chemotherapies. Due to their proven druggability, G protein-coupled receptors (GPCRs) are promising targets for anthelmintics. However, to identify candidate receptors, a deeper understanding of GPCR signalling in schistosome biology is essential. Comparative transcriptomics of paired and unpaired worms and their gonads revealed 59 differentially regulated GPCR-coding genes putatively involved in neuronal processes. In general, the diversity among GPCRs and their integral membrane topology make it difficult to characterize and deorphanize these receptors. To overcome existing limitations, we performed a pilot approach and utilized the innovative Membrane-Anchored Ligand And Receptor yeast two-hybrid system (MALAR-Y2H) to associate potential neuropeptide ligands with their cognate receptors. Here, we demonstrated the ability to express full-length GPCRs of *Schistosoma mansoni* in a heterologous yeast-based system. Additionally, we localized GPCRs and chimeras of neuropeptides fused to the WBP1 transmembrane domain of yeast to the plasma membrane of yeast cells. Reporter gene assays indicated ligand-receptor binding, which allowed us to identify certain neuropeptides as potential ligands for two GPCRs, which had been found before to be differentially expressed in schistosomes in a pairing-dependent manner. Thus, the MALAR-Y2H system appears suitable to unravel schistosome GPCR–ligand interactions. Besides its relevance for understanding schistosome biology, identifying and characterizing GPCR–ligand interaction will also contribute to applied research aspects.

## Introduction

Schistosomiasis is a neglected tropical disease caused by platyhelminths of the genus *Schistosoma*. The disease has global impact on human and animal health. According to the WHO, approximately 600 million people live in endemic areas, of which >200 million require treatment with a mortality rate of ~24 000 people in 2016 (WHO Global, 2016). Schistosomes belong to the few members of platyhelminths that have evolved separate sexes (Beltran and Boissier, 2008), and they exhibit a unique reproductive biology because the female's sexual maturation depends on a constant pairing-contact with the male (Kunz, 2001; LoVerde, 2002). Currently medical treatment is based upon a single drug, praziquantel. Although it is safe, affordable and effective, its widespread use for mass treatment has increased the likelihood of resistant parasites (Bergquist *et al*., 2017). This requires the development of alternative antischistosomal compounds.

G protein-coupled receptors (GPCRs) are the largest known superfamily of membrane receptors with a seven-transmembrane domain, and they typically interact with ligands in the extracellular domain and with heterotrimeric GTP binding proteins (G proteins) at intracellular domains. GPCRs are phylogenetically classified into five families, Glutamate, Rhodopsin, Adhesion, Frizzled/Taste2, and Secretin (Schiöth and Fredriksson, 2005). Neuropeptides typically activate receptors from the Rhodopsin family (class A receptors). Upon ligand binding, a GPCR undergoes conformational changes and releases membrane-associated G-proteins (subunits *α*, *β* and *γ*). In this state, the activated GPCR acts as a guanine nucleotide exchange factor by inducing GTP (guanosine triphosphate) exchange in the *α* subunit and its consequent dissociation from the *βγ* dimer; both dissociated *α* and *βγ* subunits can elicit downstream effects (Frooninckx *et al*., 2012).

The neuropeptidergic signalling component of invertebrate nervous systems is highly developed and complex. The system involves many different neuropeptide components, the neuropeptide processing enzymes, the endogenous neuropeptide receptors and further molecules involved in signalling cascades. Neuropeptide maturation occurs in a modulated fashion in helminths compared to vertebrates (Mair *et al*., 2004; Atkinson *et al*., 2010); a single gene can encode multiple copies of the same or different neuropeptides, and the precursor molecule undergoes extensive posttranslational processing in the trans-Golgi network prior to the production of individual neuropeptides. The processing in trematodes involves three major steps, (1) digestion of a precursor protein by a prohormone convertase (PC) at specific dibasic amino acid motifs (often two consecutive residues of lysine and arginine), (2) digestion of the carboxyterminal amino acid (aa) of the peptide-processing intermediates by a carboxypeptidase E (CPE), and (3) the amidation of the glycine C-termini in two sequential steps by peptidylglycine-*α*-hydroxylating monooxygenase (PHM) and peptidyl-*α*-hydroxyglycine *α*-amidating lyase (PAL) (McVeigh *et al*., 2012).

In *Schistosoma mansoni*, 47 potential neuropeptides emerging from 32 neuropeptide precursor (npp) genes have been identified by *in silico* analysis (McVeigh *et al*., 2009; Koziol *et al*., 2016). Furthermore, RNAseq analysis provided a comprehensive dataset of genes that are differentially transcribed in paired and unpaired schistosome males and females (Lu *et al*., 2016). Among these were 27 *Sm_npps*, and all major subfamilies of GPCRs including a platyhelminth-specific rhodopsin subfamily (Zamanian *et al*., 2011; Campos *et al*., 2014; Hahnel *et al*., 2018). Interestingly, transcript levels of almost all *Sm_npps* and many GPCRs are highest in males (paired or unpaired) and also in unpaired females. This suggested lower importance of neuronal processes in the female after pairing (Lu *et al*., 2019).

Our knowledge of the biological meaning of neuronal signalling *via* GPCRs is still fragmentary in schistosomes. Unravelling these processes would help to understand the biology of the parasite and at least part of the interplay between males and females. Towards this end, the identification of endogenous ligands for trematode peptidergic GPCRs (deorphanization) is one step needed to enable the functional characterization of GPCRs.

The primary aim of this work was to find a way to deorphanize *S. mansoni* GPCRs by employing the Membrane-Anchored Ligand And Receptor yeast two-hybrid system (MALAR-Y2H) system. As a state of the art method, this system broadens the spectrum of technical possibilities to identify protein–protein interactions (PPIs) of transmembrane receptors and extracellular ligands (Li *et al*., 2016). To achieve this goal we generated a library of 47 neuropeptides fused *via* a flexible linker to the transmembrane domain (TMP) of yeast WBP1 (te Heesen *et al*., 1991). The intracellular part of these fusion constructs consisted of the C-terminal part of ubiquitin (C_ub_) and the transcription factor GAL4. The N-terminal part of ubiquitin (N_ub_G) is joined to another construct harbouring the potential interaction partner, a GPCR. Interaction of ligands and receptors initiate a cascade that ultimately liberate the transcription factor GAL4 leading to reporter gene activation. Here, we present the full-length expression of seven *S. mansoni* GPCRs in a heterologous yeast system. One of these coding sequences (CDS) had to be codon-optimized to meet the requirements of the yeast 3′ processing machinery during transcription (Parker-Manuel *et al*., 2015). Furthermore, we detected fusions of (1) ligands with TMP and (2) receptors, linked on the C-terminus with GFP, and localized these to the plasma membrane of yeast cells. The results of the MALAR-Y2H assay suggested neuropeptide-receptor interaction of two GPCRs, which allowed us to identify potential ligands.

## Materials and methods

### Plasmid constructs

Based on previous work, we generated bait plasmid pGAD SP-WBP1_cloning_linker_TMP_C_ub__ GAL4 by cutting pGAD WBP1-C_ub_-GAL4 (Li *et al*., 2016) with *Kpn*I and *Mlu*I and assembled it with a gBlock^®^ (IDT, Iowa, USA) to destroy *Acc*65I restriction site. The intermediate clone was digested with *Nco*I, and a second gBlock was introduced with the following modifications: (1) WBP1 aa 24-430 was deleted, (2) a flexible linker was introduced 5′ to TMP, and (3) new *Acc*65I and *Sma*I restriction sites were generated to insert neuropeptides in frame with the WBP1 signal-peptide aa 1-23.

The neuropeptide library was generated by cutting pGAD SP-WBP1_cloning_linker_TMP_C_ub__ GAL4 with *Acc*65I and *Sma*I, and by ligating annealed primers (Supplementary Table 1) with corresponding 3′ and 5′ overhangs. pGAD SP-WBP1_cloning_linker_TMP_eGFP for subcellular localization studies was constructed by cutting pGAD SP-WBP1_cloning_ linker_TMP_C_ub__GAL4 with *Sac*I and *Hind*II to remove C_ub_ and GAL4. The coding sequence for eGFP was introduced using Gibson Assembly (NEB, MA, USA) and accordingly, neuropeptides were cloned by primer annealing.

Prey plasmids were created by cutting pGBKT7 OST1-N_ub_G with *NcoI* and *NotI* and assembled it with a gBlock utilizing Gibson Assembly. The resulting vector pGBKT7_SP_OST1_ cloning_N_ub_G had the following modifications: (1) OST1 aa 28-481 were deleted retaining the SP of OST1, (2) replacement of existing *Nco*I by the same restriction site after SP of OST1, (3) introducing a *Sma*I restriction site in frame 5′ to N_ub_G. The CDS of GPCR-coding genes were amplified, using Q5^®^ polymerase, from cDNA obtained from total RNA preparation of *S. mansoni*. Primers were designed to assemble products into pGBKT7_SP_OST1_cloning_N_ub_G cut with *NcoI* and *SmaI.* For subcellular localization studies, N_ub_G was replaced by eGFP.

### Cell lines

*Escherichia coli* strain NEB^®^ 10-beta was used to clone and amplify plasmid DNA. Yeast strains used for mating-assay were AH109 (MATa, trp1-901, leu2-3, 112, ura3-52, his3-200, gal4Δ, gal80Δ, LYS2::GAL1UAS-GAL1TATAHIS3, GAL2UAS-GAL2TATA-ADE2, URA3::MEL1 UASMEL1TATA-lacZ, MEL1) and Y187 (MAT*α*, ura3-52, his3-200, ade2-101, trp1-901, leu2-3, 112, gal4Δ, met–, gal80Δ, URA3::GAL1UAS-GAL1TATA-lacZ) from Clontech (Matchmaker^®^ Y2H-system).

### RNA isolation, cDNA synthesis and polymerase chain reaction (PCR)

Rna was isolated from adult schistosomes or yeast clones with the Monarch^®^ Total RNA Miniprep Kit (NEB) and transcribed into cDNA using ProtoScript^®^ II reverse transcriptase (NEB). Both steps were carried out following the manufacturer instructions. PCR analysis for cloning full-length GPCR coding sequences and expression in yeast was performed with primer pairs as indicated in Supplementary Table 2. In brief, amplification was done with Q5 High-Fidelity DNA polymerase in a total volume of 25 *μ*L for 32 cycles (95°C for 8 s, 58°C for 20 s and 72°C for 100 s). Amplified PCR products were resolved by agarose gel electrophoresis.

### Yeast transformation and mating

Transformation of *Saccharomyces cerevisiae* yeast strains was performed according to Tripp *et al*. (2013) utilizing an optimized LiAc-method. Prey plasmids were transformed into Y187 strain and bait plasmids were transformed into AH109 strain. Transformed yeasts were selected by plating on synthetic dropout medium (SD) lacking tryptophane (SD/Trp^−^) or leucine (SD/Leu^−^), respectively. Mating was achieved through resuspending 10 *μ*L of each AH109 and Y187 clone in 500 *μ*L YPDA medium and cultivated for 16 h at 30°C. Hybrids carrying both plasmids were selected on SD/Trp^−^ Leu^−^ plates. Growth assay to monitor PPI was carried out by plating a dilution series (OD_600_ = 1, 0.1 and 0.01) of two mated yeast clones (*n* = 2) on SD/Trp^−^ Leu^−^ His^−^ Ade^−^. Interaction was documented after 72 h at 30°C by scanning the plates.

### *β*-Galactosidase assay

Colonies of mated yeasts were cultivated in SD/Trp^−^ Leu^−^; medium until OD_600_ of 0.4–0.8 was reached. One millilitre culture medium was centrifuged, and pelleted cells were resuspended in 400 *μ*L Z-buffer (60 mm Na_2_HPO_4_, 40 mm NaH_2_PO_4_, 10 mm KCl, 1 mm MgSO_4_, pH 7.0). Cells were lysed by three consecutive freeze/thaw cycles in liquid nitrogen. The lysate was mixed with 200 *μ*L Z buffer containing 0.4% o-nitrophenyl-*β*-d-galactopyranoside (ONPG) and incubated for 30 min at 30°C. After centrifugation, the absorbance of the supernatant at 405 nm was measured and Miller Units were calculated according to the equation: Miller Units = 1000 × OD_405_/*t*(min) × OD_600_.

### Confocal laser scanning microscopy

For localization analysis of receptors and neuropeptides by confocal laser scanning microscopy (CLSM), yeast cells were transfected with GFP fusion constructs and stained with the membrane specific dye Dil (Merck KGaA, Germany). In brief, cells were fixed for 10 min on ice with 4% paraformaldehyde dissolved in PBS. After two washing steps, cells were incubated with 10 *μ*m Dil and washed four times with PBS. Finally, yeasts were dried and mounted with Roti^®^-Mount FluorCare (Carl Roth, Germany). Stained yeast cells were captured on an inverse CLSM (Leica TSC SP5; Leica, Germany). GFP was excited with an argon-ion laser at 488 nm and Dil at 561 nm.

### Maintenance of the schistosome life cycle

A Liberian strain (Bayer AG, Monheim) of *S. mansoni* was used to infect freshwater snails of the genus *Biomphalaria glabrata* as an intermediate host and Syrian hamsters (*Mesocricetus auratus*) as final host (Grevelding, 1995). Eight-week-old hamsters were infected by the ‘paddling method’ (Dettman *et al*., 1989), and sacrificed at 46 days post infection to collect adult worm couples by perfusion. Worms were cultured in M199 medium [Sigma-Aldrich, Germany; supplemented with 10% Newborn Calf Serum, 1% HEPES (1 M) and 1% ABAM-solution (10 000 units penicillin, 10 mg streptomycin and 25 mg amphotericin B per mL)] at 37°C and 5% CO_2_.

### Ethical standard

Animal experiments were performed in accordance with the European Convention for the Protection of Vertebrate Animals used for experimental and other scientific purposes (ETS No 123; revised Appendix A) and were approved by the Regional Council (Regierungspraesidium) Giessen (V54-19 c 20/15 c GI 18/10).

## Results

### Modulation of the MALAR-Y2h system

**Fig. 1.**
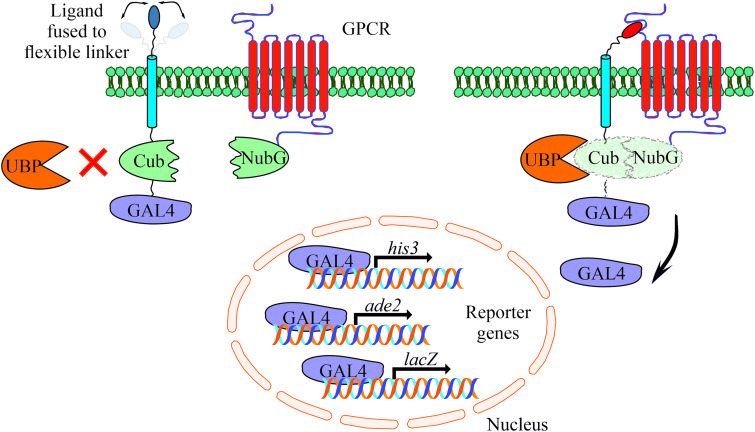
Schematic representation of the yeast-based MALAR system for detecting interactions of extracellular ligands with their potential membrane receptors. The ligands are expressed as fusion proteins and consist of a flexible linker directed to the extracellular space, a transmembrane domain, the C-terminal part of ubiquitin (C_ub_), and the GAL4 transcription factor. Receptors are fused to the N-terminal part of ubiquitin (N_ub_G) harbouring a single point mutation (Ile13Gly) to prevent spontaneous reconstitution. These vectors complement leucine and tryptophan auxotrophs, respectively. An interaction of ligand and receptor forces the split ubiquitin halves into close proximity. A ubiquitin-specific protease (UBP) recognizes the reconstituted ubiquitin, which is subsequently degraded leading to the release of GAL4. The latter enters the nucleus activating the reporter genes *his3*, *ade2* and *lacZ* to allow cell growth on selective agar plates (SD/Leu^−^ Trp^−^ His^−^ Ade^−^).

The MALAR-Y2H system is a further development of the split ubiquitin method to detect protein–protein interactions between two membrane proteins (Stagljar *et al*., 1998; Li *et al*., 2016). Y2H analysis contributed significantly to identify interacting proteins in the past with the limitation to cytosolic and membrane-bound proteins. The MALAR-Y2H method expands these applications through a chimeric transmembrane protein allowing the analysis of membrane-bound proteins and their putative interactions. In this split ubiquitin-based system, one potential interaction partner is expressed as a chimeric transmembrane protein, of which one part is an extracellular ligand fused *via* a transmembrane domain to an intracellular part. The latter consists of C_ub_ and the transcription factor GAL4. The other potential interaction partner is the receptor of interest that is fused to N_ub_G. The principle of the MALAR-Y2H system to identify PPIs is the reconstitution of the two ubiquitin halves, which is triggered by ligand–receptor interaction. Only re-formed ubiquitin can be recognized and cleaved by an endogenously expressed ubiquitin-specific protease (UBP). As a consequence GAL4 is released and transported to the nucleus to induce reporter gene expression (Fig. 1).

To further improve and adapt the system to our requirements, we introduced a flexible linker between the transmembrane domain and the neuropeptide (Argos, 1990). Most of the mature neuropeptides of schistosomes are between four and twenty amino acids in size (Koziol *et al*., 2016), thus they represent relatively small molecules compared to classic chemokines like the CXC-motif chemokine ligand 12 (CXCL12) that was used in the study of Li *et al*. (2016). Flexible linkers consist of relatively small amino acids such as glycine, serine and threonine and often occur in natural proteins. Besides their flexibility, they are polar to form hydrogen bonds with water helping to maintain the stability of the linker structure in aqueous solvents. This may prevent steric hindrance due to a rigid structure of the chimeric transmembrane protein, and we assumed that this may facilitate ligand–receptor interaction.

### Full-length expression of *S. mansoni* GPCRs in *S. cerevisiae*

As shown in a previous study, AT-rich DNA sequences of *S. mansoni* genes can be misinterpreted by the yeast 3′ processing machinery during transcription as cleavage and poly-adenylation signals, which leads to truncated transcripts (Parker-Manuel *et al*., 2015). To overcome this limitation, a computer program was developed for the prediction of mRNA 3′ processing sites by providing a discrete state-space (DSM) value (Graber *et al*., 2002). A higher DSM value indicates a higher probability of a 3′ processing site at a specific DNA sequence. For *S. mansoni α* integrins 2, 3 and 4 it was shown that DSM values >14 exhibited a 3′ processing signal leading to truncated proteins (Parker-Manuel *et al*., 2015). We addressed this by examining full-length transcription after cloning GPCR coding sequences into a yeast expression vector, which were transformed into strain Y187. Total RNA was isolated and reverse transcribed into cDNA (+RT) followed by a PCR using specific primers for amplifying full-length transcripts of respective GPCRs.

Of seven GPCR-coding genes, only for one (Smp_244240) no amplification product was obtained using gene-specific primers (data not shown). This correlated with maximum DSM values up to 20.58 for this gene. PCR analyses with three different primer pairs amplifying the 5′-proximal region of Smp_244240 (Supplementary Fig. 1A) resulted in an amplification product using reverse primer 1 (rev1) only in one out of several yeast clones tested. No products were obtained using other primers rev2 or rev3. It appeared that the mRNA was prematurely truncated, probably between base pairs 190 and 301. This assumption coincided with the highest DSM peaks for Smp_244240, which occurred between base pairs 200 and 350 (Supplementary Fig. 1B). Next, we applied the Java codon adaption tool (JCAT) as described before (Parker-Manuel *et al*., 2015) to optimize the coding sequence without affecting the amino acid sequence. The resulting product was sequenced to confirm identity before subsequent cloning into the yeast expression vector.

**Fig. 2.**
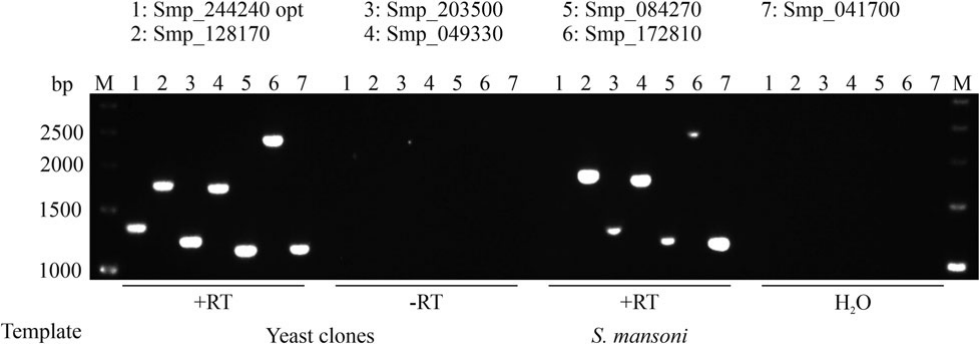
Full-length expression of *S. mansoni* GPCRs in *S. cerevisiae*. Wild type or optimized CDSs of GPCRs were transformed into yeast strain AH109. RNA was isolated and transcribed into cDNA. Specific primer pairs were used to amplify corresponding GPCRs (numbers 1-7) by RT-PCR, and resulting amplicons were size-separated on a 1% agarose gel (M = marker, bp = base pair). The absence of reverse transcriptase (-RT) during cDNA synthesis or cDNA synthesis without template (H2O) served as negative controls. RNA isolated from *S. mansoni* served as a positive control for full-length transcripts. In each case, amplicons of the expected sizes were obtained.

The optimized sequence (Smp_244240 opt) and the other six GPCRs (DSM scores <13) tested showed full-length transcripts in yeast (Fig. 2). No signal was detected in the absence of reverse transcriptase (−RT), which excluded DNA contamination of the RNA samples. Reverse transcribed RNA isolated from *S. mansoni* showed the same size of amplicons as demonstrated for the yeast clones. These results suggested that full-length transcripts of these GPCRs were present in yeast.

### Detecting plasma membrane localization of heterologous GPCRs in *S. cerevisiae*

**Fig. 3.**
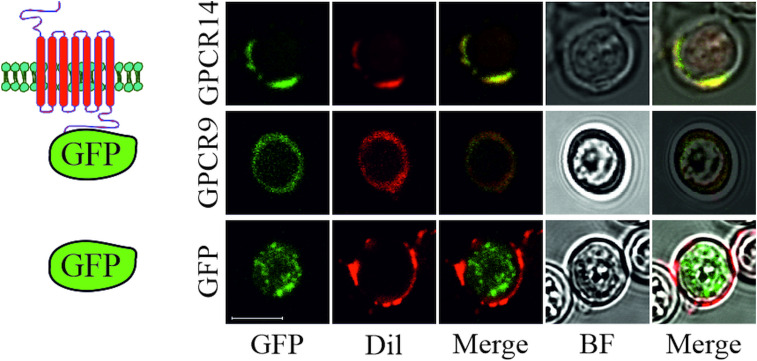
Subcellular localization of *S. mansoni* GPCRs in *S. cerevisiae*. Plasmids coding for eGFP or GPCRs (Smp_203500 (GPCR14) and Smp_244240opt (GPCR9)) tagged with eGFP at the C-terminus were transformed into yeast strain Y178. Fluorescence of eGFP and the membrane-stain Dil were recorded by CLSM. Fluorescent images were merged individually or with bright field (BF) images, respectively. Scale bar, a representative for all pictures: 5 *μ*m.

To reveal the cellular localization of recombinant *S. mansoni* GPCRs guided by the signal peptide of OST1 (yeast OST1, residues 1–27) in yeast, we tagged selected GPCRs at their C-terminus with eGFP. Microscopic expression analysis revealed that GPCR9 (Smp_244240) and GPCR14 (Smp_203500) were predominantly located in the membrane as visualized by an overlay with the membrane-specific dye Dil (Honig and Hume, 1986) (Fig. 3). In contrast, the fluorescent signals for GPCR13-GFP (Smp_128170) were mainly detected in the cytoplasm (Supplementary Fig. 2B), as expected for the expression of GFP without fusion partner. Furthermore, besides their cytoplasmic occurrence, we confirmed that fusion proteins of a representative neuropeptide (NPP5a), fused to the transmembrane domain WBP1 (yeast WBP1, residues 341–430) and guided by the signal peptide of WBP1 (residues 1–23), was also targeted to the outer membrane (Supplementary Fig. 2A).

### Monitoring *S. mansoni* GPCR and neuropeptide interactions

Previous work described the establishment of the MALAR-Y2H system to detect PPIs between secreted and transmembrane proteins (Li *et al*., 2016). The ligand CXCL12 and the mouse receptor CXCR4, representing a well-studied chemokine-signalling pathway, served as model interacting partners in this study.

**Fig. 4.**
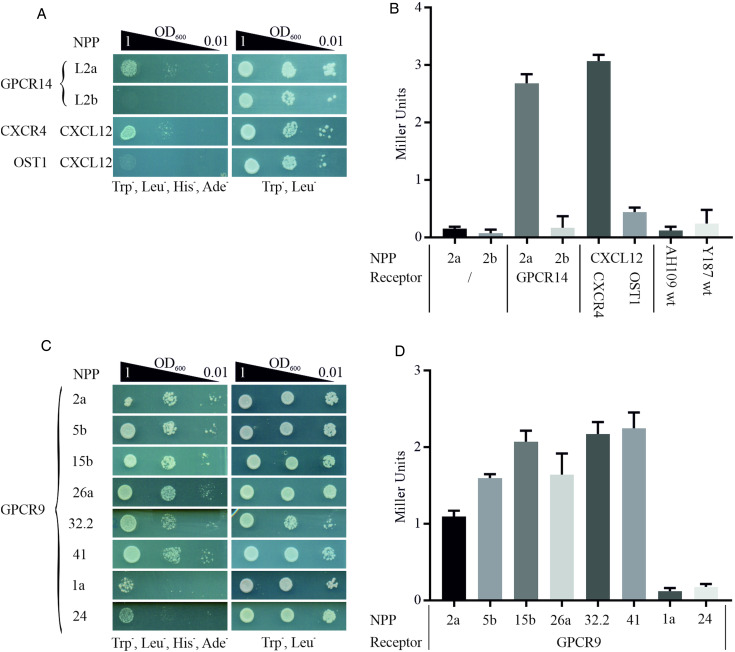
Detection of protein–protein interactions between GPCRs and hormone peptides. Shown are cell growth assays of yeast strain AH109 transfected with plasmids expressing ligand fusion proteins (NPP), which was mated with yeast strain Y187 transfected with plasmids expressing (A) Smp_203500 (GPCR14) and (C) Smp_244240opt (GPCR9), respectively. The classic chemokine CXCL12 and its known receptor CXCR4 were employed as a positive control (Li *et al*., 2016). OST1 is a transmembrane protein of *S. cerevisiae* and in combination with CXCL12 used as the negative control. Three different OD_600_ concentrations of diploid yeast cells were dropped onto SD/Trp^−^ Leu^−^ His^−^ Ade^−^ and Trp^−^ Leu^−^ plates; the latter served as growth control. Colony growth was monitored after 72 and 48 h, respectively. (B, D) ONPG-assays to measure *β*-Gal activity of diploid cells as in A and C. Cells were lysed in liquid nitrogen and incubated with ONPG for ~30 min at 30°C. OD_405_ was measured, and Miller Units were calculated (Miller Units = 1000 × OD_405_/*t*(min) × OD_600_). Shown are the mean values of two clones (*n* = 2).

We employed this system to start deorphanizing *S. mansoni* GPCRs and therefore generated a library of 47 neuropeptides (Supplementary Table 3) as chimeric proteins fused *via* a flexible linker to the C-terminal part of the split ubiquitin system. In analogy, GPCRs were cloned as fusion proteins with the N-terminal part of ubiquitin. Mated clones were selected on SD/Trp^−^ Leu^−^ plates to ensure co-expression of bait and prey in the cells (Fig. 1). The combination of receptor GPCR14 and NPP2a revealed growth on SD/Trp^−^ Leu^−^ His^−^ Ade^−^ agar plates. Only weak growth was monitored for all other 46 combinations (exemplified by NPP2b). This suggested a specific interaction of GPCR14 with NPP2a (Fig. 4A). Growth on SD/Trp^−^ Leu^−^ plates showed only slight differences between all combinations indicating that clonal effects were not responsible for our finding. The positive control (CXCR4/CXCL12) showed growth comparable to GPCR14/NPP2a and the negative control (OST1/CXCL12) to all other tested ligands supporting the functionality of the assay. In addition, we investigated the *β*-Gal activity of the hybrid cells. The results were consistent with the growth assays. Combinations of GPCR14/NPP2a and CXCR4/CXCL12 revealed Miller units of 2.68 and 3.07, respectively (Fig. 4B). Yeast strain AH109 transfected only with bait plasmids NPP2a and NPP2b showed Miller units comparable with the wild-type strain indicating no self-activation or a false positive release of the GAL4 transcription factor. The transcript levels of *gpcr14* and *npp2* have been shown to be highest in males and unpaired females supporting the possibility of genuine interactors (Supplementary Fig. 3).

With GPCR9 as prey, we observed the strongest growth with ligands NPP2a, 5b, 15b, 26a, 32.2 and 41 on SD/Trp^−^ Leu^−^ His^−^ Ade^−^ agar plates (Fig. 4C). The weakest growth was detected for ligands NPP1a and NPP24. The remaining 39 hybrids showed a weak to moderate growth in the range of OD_600_ 1 and 0.1 (data not shown). All tested clones showed equal growth on SD/Trp^−^ Leu^−^ agar plates. The results of the *β*-Gal assays were in line with the growth assays (Fig. 4D). Miller Units ranged from 1.09 to 2.25 for strongly growing hybrids, and 0.12 and 0.17 for GPCR9/NPP1a and GPCR9/NPP24, respectively. RNA transcripts of *gpcr9* have been solely detected in testis of males (Supplementary Fig. 3). Transcript levels of *npp5*, *2* and *41* were highest in males and unpaired females, whereas *npp15*, *26* and *32.2* were most prominent in unpaired females. Furthermore, we conducted the MALAR-Y2H assay with the ligand library and GPCR13. In contrast to GPCR9 and GPCR14, we only monitored weak to no growth on SD/Trp^−^ Leu^−^ His^−^ Ade^−^ agar plates with all 47 hybrids (Supplementary Fig. 4).

## Discussion

Our current knowledge about PPI networks has been largely derived from Y2H analysis (Suter *et al*., 2008). The original method was developed to investigate PPI of cytoplasmic proteins (Fields and Song, 1989). This groundwork paved avenues for more sophisticated applications to detect PPIs between cytoplasmic proteins and membrane proteins, and between two membrane-associated proteins (Stagljar *et al*., 1998). A further modification, MALAR-Y2H system, even allowed the detection of PPIs on the outer cytoplasmic membrane between secreted cytokines and transmembrane receptors (Li *et al*., 2016). We have employed the MALAR-Y2H system to identify PPIs of GPCRs with their putative neuropeptides. Although yeasts are eukaryotic organisms, possessing the necessary machinery for post-translational processing, it is not self-evident that genes from *S. mansoni* are processed correctly in the heterologous yeast system. Indeed, previous experiments with *S. mansoni α*-integrins (Parker-Manuel *et al*., 2015) and findings from this study showed that sequence structure influences transcription. One out of seven tested CDS of *S. mansoni* GPCRs was not expressed as a full-length transcript. As in the previous study (Parker-Manuel *et al*., 2015), this correlated with a DSM-score of >20, which indicates a recognition sequence of the 3′ mRNA processing machinery of yeast (Graber *et al*., 2002) interfering with the transcription of this GPCR-coding gene. As DSM output, a score indicated the probability of a 3′-processing site being present and activated at that particular cleavage point within this sequence. To overcome this limitation, we optimized the codon sequences without disturbing the aa sequence. Subsequently, we succeeded in expressing the optimized version of Smp_244240opt (GPCR9). Using yeast as a model for gene heterologous gene expression could also affect molecular work with other parasites with a high AT content such as *Plasmodium* sp. (Gardner *et al*., 2002; Videvall, 2018).

Using the MALAR-Y2H system, we assumed that schistosome neuropeptides and receptors are transported and integrated into the plasma membrane. This is facilitated by a secretory signal peptide (SP) located at the N-terminus of the fusion protein. This SP usually contains an N-terminal basic aa followed by a stretch containing hydrophobic residues (Heijne, 1990). During protein maturation, the SP is cleaved off. We used the SPs of yeast WBP1 and OST1 for neuropeptides and GPCRs, respectively. It has been demonstrated before that these SPs are feasible to target receptors and ligands to the membrane (Li *et al*., 2016). Additionally, a prediction tool supported the presence of signal peptides and their cleavage sites in all chimeric proteins used in our study (Almagro Armenteros *et al*., 2019). In this pilot approach, we investigated the localization of ligands and GPCRs. As assumed, we observed that ligand fusion proteins such as the representative neuropeptide (L5a) that had been fused to the transmembrane domain of WBP, was targeted to the outer membrane. This corresponds to previous data with the chemokine ligand CXCL12 using the same system (Li *et al*., 2016) confirming the suitability of this approach also for schistosome neuropeptides. For GPCRs, we observed that GPCR9 and GPCR14 localized to the plasma membrane. This was confirmed by a ring-shaped GFP signal, indicating membrane localization and co-localization with a membrane-specific dye. Unexpectedly, the fluorescence signal for GPCR13 was mainly localized in the cytoplasm. In this case, the failure of membrane localization could have different reasons. First, we observed a lack of growth of GPCR13 in combination with all tested neuropeptides. Thus the combination of the chosen SP and the receptor may not have matched and recognized by yeast cells leading to failure of membrane trafficking. To improve GPCR13 membrane trafficking, combinations with different SPs could be tested. Recently, synthetic SPs were developed that enable efficient secretory protein production in yeast that could help in this respect (Yarimizu *et al*., 2015). Second, some receptors have been reported to be poorly expressed because they get stuck in the membrane of the endoplasmic reticulum, or they are directed to the vacuoles undergoing proteolytic degradation (Reiländer, 1998). Finally, although it appears unlikely to us, we cannot finally exclude that GPCR prediction by bioinformatics (Berriman *et al*., 2009; Hahnel *et al*., 2018) may have resulted in few false-positive results.

Using the MALAR-Y2H system as a novel approach to deorphanize schistosome GPCRs investigating neuropeptide interactions led to first promising results. An obvious advantage of this system is that GPCRs and ligands are co-expressed at the membrane which favours physical proximity as a prerequisite for interaction. This is especially the case for longer neuropeptides, if tertiary structure formation is required for proper interaction (Keire *et al*., 2002). Here, the addition of a linker may be beneficial allowing a higher spatial degree of freedom. In addition, the receptor does not have to be ‘activated’ in the sense of conformational change and G-protein recruitment. A higher degree of spatial freedom may also explain why more than one interaction was found in the case of GPCR9. Although it is well established that GPCRs can interact with more than one specific ligand and vice versa (Luttrell, 2008), we cannot completely rule out that not all detected neuropeptides are genuine ligands also *in vivo.* In the heterologous system, ligands are faced with GPCRs, and they may interact based on structural prerequisites. However, it may also be possible that they may not face each other *in vivo* due to expression in e.g. different tissues or at different time points during development. In addition, the neuropeptides do not undergo a C-terminal amidation, which might be a source of undetected interactions. On the other hand, we detected only one potential ligand (NPP2a) for GPCR14. This finding proves that the system can be selective and leakiness appears to be low since yeast cells only transfected with bait plasmids (NPP2a & NPP2b) showed low Miller Units comparable to the wild type yeast strain AH109. The latter indicated that the C-terminal part of ubiquitin alone is not cleaved by UBP, which prevents false-positive results. In conclusion, the PPI of receptor and ligand was the driving force to ultimately release GAL4 for activating reporter genes.

To substantiate indicative findings of PPIs identified by MALAR-Y2H system, further approaches are needed. One possibility to identify natural ligands of GPCRs is to take advantage of the promiscuous nature of the G-protein subunit G*α*_16_ that couples a wide range of GPCRs to the phospholipase C-*β* pathway. Co-expression of G*α*_16_ along with GPCRs of interest and mitochondrially targeted apoaequorin in CHO cells enable sensitive monitoring of intracellular calcium responses to exogenous ligands. This way the planarian neuropeptide NPY8 has been shown to be the cognate ligand for NPYR1 (Saberi *et al*., 2016). NPY8 plays a crucial role in sexual maturation and germ cell differentiation. Another approach is the use of the yeast pheromone signalling pathway for GPCR-ligand screening (Klein *et al*., 1998; Minic *et al*., 2005). This requires the development of yeast strains containing a chimeric G*α* subunit composed of segments that couple to mammalian GPCRs and yeast segments that interact with the G*β*/G*γ* subunits. Hence, an outer signal can be coupled to the pheromone response signalling cascade activating an exogenous reporter gene (in most cases complementing *his3* auxotrophs). These procedures have two potential benefits compared to the MALAR-Y2H system. These are free-floating neuropeptides including the possibility for a C-terminal amidation. It has been demonstrated that this modification is essential for biologic activity (Eipper *et al*., 1992; McVeigh *et al*., 2011). Furthermore, activation of the receptor by its natural ligand can be monitored in a dose-dependent manner. A *S. mansoni* G protein-coupled acetylcholine receptor expressed in yeast cells showed enhanced activity when incubated with increased acetylcholine concentrations (100 nM–10 *μ*M) (MacDonald *et al*., 2015). Nevertheless, the coupling efficiency of heterologous GPCRs with chimeric G*α* subunits is a major issue when the functional response of a receptor is assessed *in vivo*. In order to use yeast for *S. mansoni* GPCR functional characterization, there could be a need for yeast genetic modifications to introduce a proper link between the heterologous receptor and the yeast signalling cascade. So far there are yeast strains available consisting of mammalian chimeric G*α* subunits. In future experiments, we intend to analyse the obtained PPI in these systems as well to further strengthen our above findings. Indeed, a *S. mansoni* dopamine receptor was shown to be responsive to dopamine in a dose-dependent manner, whereas other structurally related amines had no effect (Taman and Ribeiro, 2009). The receptor activity was highest with a chimeric G*α* gene in which the last five amino acids of yeast G*α* were replaced with those of human G*α*_S_. Lower or no receptor activity was detected with strains carrying chimeras of yeast G*α* and human G*α*_i2_, G*α*_12_, G*α*_0_ and G*α*_q_. This supports the conclusion that the proper linking of GPCRs to the genetically modified pheromone response pathway has to be carefully tested.

In summary, the data of our pilot study suggest that the MALAR-Y2H system provides a suitable way to deorphanize GPCRs of interest and to obtain first hints of their putative ligands.

## Supplementary material

For supplementary material accompanying this paper visit http://dx.doi.org/10.1017/S0031182019001756. click here to view supplementary material
