# Supplementary material for: Towards deorphanizing G protein-coupled receptors of Schistosoma mansoni using the MALAR yeast two-hybrid system
Source: Parasitology. 2019 Dec 16;147(8):865–72. doi: 10.1017/S0031182019001756 (PMC7284817; doi:10.1017/S0031182019001756)
Supplement: Supplementary file 1 [file S0031182019001756sup.zip › S0031182019001756sup001.docx]

Supplementary table 2: Primers used for GPCR gene amplification

| **GPCR** | **5’-3’ sequence (forward and reverse)** | **Length**  **(bp)** |
| --- | --- | --- |
| Smp_244240 opt. (R9) | CCGCCCAATACGAGCCCATGAACGAATCTATCATCCAAAACAACC | 45 |
|  | TGATCCACCTTCTAGGATCCCGTTAGAGTCACATTCGTTACCAACC | 46 |
| Smp_128170 (R13) | CCGCCCAATACGAGCCCATGGATCAGCTGGGTCGGAT | 37 |
|  | GTTGATCCACCTTCTAGGATCCCTTCTGGCTGTTGTGTAATAACTGAC | 48 |
| Smp_203500 (R14) | CCGCCCAATACGAGCCCATGAAATTGTATTCCTGTTGGCTG | 41 |
|  | GTTGATCCACCTTCTAGGATCCCAGCAGCTGTTTCATTATCAAAAC | 46 |
| Smp_049330 (R18) | CCGCCCAATACGAGCCCATGGGTATACTTCGAATAAGTATTTTAACC | 47 |
|  | GTTGATCCACCTTCTAGGATCCCGAGTGAACAACGATAACCCACC | 45 |
| Smp_084270 (R20) | CCGCCCAATACGAGCCCATGATAAGTATGAACTCAAGTGAATT | 43 |
|  | GTTGATCCACCTTCTAGGATCCCGTAATTGTGGCCTGATACAACG | 45 |
| Smp_041700 (R21) | CCGCCCAATACGAGCCCATGTTGAGTTTTTCATTTCATCCAAGTAGT | 47 |
|  | GTTGATCCACCTTCTAGGATCCCGTTTGATGCACTTTGAACAGGTAAAGT | 50 |
| Smp_041700 (R24) | CCGCCCAATACGAGCCCATGATATTATCGATTGACGGTAAT | 41 |
|  | GTTGATCCACCTTCTAGGATCCCTATTGGTGATGCTTGTGAACAATTC | 48 |
| Smp_244240 for1 | ATGAATGAATCAATAATACAAAATAATCAATC | 41 |
| Smp_244240 rev1 | AAGGATTTTTTAATAATGTATTCAC | 48 |
| Smp_244240 rev2 | TAGCTAATGATTTTGGATTACG | 41 |
| Smp_244240 rev3 | CACCAAACCATGCTAATGC | 48 |
