# Supplementary material for: Towards deorphanizing G protein-coupled receptors of Schistosoma mansoni using the MALAR yeast two-hybrid system
Source: Parasitology. 2019 Dec 16;147(8):865–72. doi: 10.1017/S0031182019001756 (PMC7284817; doi:10.1017/S0031182019001756)
Supplement: Supplementary file 1 [file S0031182019001756sup.zip › S0031182019001756sup002.docx]

Supplementary table 3: Amino acid sequences of all cloned neuropeptides

| **Entry** | **Gene name** | **Amino acid sequence** |
| --- | --- | --- |
| 1 | Smp_042120.1_npp-1a | AFVRLG |
| 2 | Smp_042120.1_npp-1b | GFVRIG |
| 3 | Smp_154970.1_npp-2a | RGMIG |
| 4 | Smp_154970.1_npp-2b | RGFMG |
| 5 | Smp_052880.1_npp-5a | AAYMDLPWG |
| 6 | Smp_052880.1_npp-5b | AAYIDLPWG |
| 7 | Smp_070100.1_npp-6 | AVRLMRLG |
| 8 | Smp_153070.1_npp-13 | HFMPQRFG |
| 9 | Smp_150650.1_npp-14 | GLRNMRMG |
| 10 | Smp_136760.1_npp-15a | VQFLRLG |
| 11 | Smp_136760.1_npp-15b | SAYPYVG |
| 12 | Smp_142160.1_npp-16 | NYLWDTRLG |
| 13 | Smp_088360.1_npp-20a | AQALAKLMSLFYTSDAFNKYMENLDAYYMLRGRPRFG |
| 14 | Smp_159950.1_npp-20b | AVEIVPPERPFIFETPEALRTYLHKLNEYFAIIGRPRFG |
| 15 | Smp_188580.1_npp-23 | YIRFG |
| 16 | Smp_192780.1_npp-24 | GGMYGGLLG |
| 17 | Smp_071050.1_npp-26a | TLFNPILF |
| 18 | Smp_071050.1_npp-26b | NFDPILF |
| 19 | Smp_071050.1_npp-26c | SYFDPIIY |
| 20 | Smp_071050.1_npp-26d | SYFDPILF |
| 21 | Smp_071050.1_npp-26e | EHFDPIIY |
| 22 | Smp_134550.1_npp-27 | VPPYITGGIRY |
| 23 | Smp_043650.1_npp-28 | AYHFFRL |
| 24 | Smp_202260.1_npp-29 | MVYW |
| 25 | Smp_201210.1_npp-31/32.1A | GPETLWELD |
| 26 | Smp_322590.1_npp-31/32.1B | GPEPLWVVET |
| 27 | Smp_147060.1_npp-31/32.2 | GPELIIPFISGGVPA |
| 28 | Smp_154220.1_npp-35.1 | YGHYSQRLG |
| 29 | Smp_203190.1_npp-35.2 | YYISQRLG |
| 30 | Smp_136620.1_npp-36.1 | WFPIKEYRGGLMEV |
| 31 | Smp_245940.1_npp-36.2_a | WYPVKEFHYDEPLEI |
| 32 | Smp_245940.1_npp-36.2_b | WFPVKEFHYDGPLEV |
| 33 | Smp_245940.1_npp-36.2_c | WSPVKEFHYDEPIEV |
| 34 | Smp_321760.1_npp-37 | WTDF |
| 35 | Smp_141480.1_npp-38a | VLADY |
| 36 | Smp_141480.1_npp-38b | QAILADY |
| 37 | Smp_200530.1_npp-39 | FTRPYG |
| 38 | Smp_004710.1_npp-40a | FLLALPSP |
| 39 | Smp_004710.1_npp-40b | FLLGLPPKVEH |
| 40 | Smp_004710.1_npp-40c | FLLGLPPSLRQH |
| 41 | Smp_004710.1_npp-40d | FILGLPAPTRFHS |
| 42 | Smp_200800.1_npp-41 | FFCNPMGCV |
| 43 | Smp_316910.1_npp-42 | PWTLRDPLNCCLDNAKCC |
| 44 | Smp_201600.1_npp-43a | ASLAYF |
| 45 | Smp_201600.1_npp-43b | ASLSYF |
| 46 | Smp_170740.1_npp-47 | GKFFMLG |
| 47 | Smp_161240.1_npp-48 | YYTNLKTIDWPNPLTLSEAILSEQAAIAEAASNSALPSSSSSILSRGQFIAPDVDYYPTWSESYLYLKPQRNIDEELRKLKAEPPRGLPNVMRYG |
