# Supplementary material for: Towards deorphanizing G protein-coupled receptors of Schistosoma mansoni using the MALAR yeast two-hybrid system
Source: Parasitology. 2019 Dec 16;147(8):865–72. doi: 10.1017/S0031182019001756 (PMC7284817; doi:10.1017/S0031182019001756)
Supplement: Supplementary file 1 [file S0031182019001756sup.zip › S0031182019001756sup004.docx]

Supplementary table 1: Primers used to generate neuropeptide CDS

| **NPP** | **5’3’ sequence (forward and reverse)** | | **Length**  **(bp)** |
| --- | --- | --- | --- |
| 1a | GTACCCAAACCGCTTTTGTACGTCTGGGG | | 29 |
|  | CCCCAGACGTACAAAAGCGGTTTGG | | 25 |
| 1b | GTACCCAAACCGGATTTGTTCGGATCGGT | | 29 |
|  | ACCGATCCGAACAAATCCGGTTTGG | | 25 |
| 2a | GTACCCAAACCCGAGGAATGATTGGC | | 26 |
|  | GCCAATCATTCCTCGGGTTTGG | | 22 |
| 2b | GTACCCAAACCCGAGGTTTTATGGGT | | 26 |
|  | ACCCATAAAACCTCGGGTTTGG | | 22 |
| 5a | GTACCCAAACCGCTGCTTACATGGATTTACCATGGGGT | | 38 |
|  | ACCCCATGGTAAATCCATGTAAGCAGCGGTTTGG | | 34 |
| 5b | GTACCCAAACCGCAGCTTATATTGATTTACCATGGGGT | | 38 |
|  | ACCCCATGGTAAATCAATATAAGCTGCGGTTTGG | | 34 |
| 6 | GTACCCAAACCGCTGTCCGATTAATGAGACTTGGT | | 35 |
|  | ACCAAGTCTCATTAATCGGACAGCGGTTTGG | | 31 |
| 13 | GTACCCAAACCCATTTTATGCCTCAACGATTTGGA | | 35 |
|  | TCCAAATCGTTGAGGCATAAAATGGGTTTGG | | 31 |
| 14 | GTACCCAAACCGGATTACGTAATATGCGTATGGGT | | 35 |
|  | ACCCATACGCATATTACGTAATCCGGTTTGG | | 31 |
| 15a | GTACCCAAACCGTTCAATTTCTACGTCTTGGT | | 32 |
|  | ACCAAGACGTAGAAATTGAACGGTTTGG | | 28 |
| 15b | GTACCCAAACCTCTGCTTATCCTTATGTTGGT | | 32 |
|  | ACCAACATAAGGATAAGCAGAGGTTTGG | | 28 |
| 16 | GTACCCAAACCAATTATTTATGGGATACACGTTTGGGT | | 38 |
|  | ACCCAAACGTGTATCCCATAAATAATTGGTTTGG | | 34 |
| 20a | Fw1 | GTACCCAAACCGCACAAGCATTAGCTAAACTTATGTCATTATTTTATACTAGTGATGCAT | 60 |
|  | Fw2 | TTAATAAATATATGGAAAATCTTGATGCATATTATATGCTTAGAGGTAGACCAAGATTTGGT | 62 |
|  | Re1 | TTAAATGCATCACTAGTATAAAATAATGACATAAGTTTAGCTAATGCTTGTGCGGTTTGG | 60 |
|  | Re2 | ACCAAATCTTGGTCTACCTCTAAGCATATAATATGCATCAAGATTTTCCATATATTTA | 58 |
| 20b | Fw1 | GTACCCAAACCGCAGTTGAAATTGTTCCACCAGAAAGACCATTTATATTTGAAACACCTG | 60 |
|  | Fw2 | AAGCTCTTAGAACATATTTACATAAATTAAATGAATATTTTGCTATTATAGGTCGTCCTAGATTTGGT | 68 |
|  | Re1 | GCTTCAGGTGTTTCAAATATAAATGGTCTTTCTGGTGGAACAATTTCAACTGCGGTTTGG | 60 |
|  | Re2 | ACCAAATCTAGGACGACCTATAATAGCAAAATATTCATTTAATTTATGTAAATATGTTCTAAGA | 64 |
| 23 | GTACCCAAACCTATATTAGATTTGGA | | 26 |
|  | TCCAAATCTAATATAGGTTTGG | | 22 |
| 24 | GTACCCAAACCGGTGGAATGTATGGTGGTCTATTAGGA | | 38 |
|  | TCCTAATAGACCACCATACATTCCACCGGTTTGG | | 34 |
| 26a | GTACCCAAACCAATTTTGATCCAATTCTGTTT | | 32 |
|  | AAACAGAATTGGATCAAAATTGGTTTGG | | 28 |
| 26b | GTACCCAAACCTCATACTTTGATCCAATTATTTAT | | 35 |
|  | ATAAATAATTGGATCAAAGTATGAGGTTTGG | | 31 |
| 26c | GTACCCAAACCTCATACTTTGATCCTATATTATTT | | 35 |
|  | AAATAATATAGGATCAAAGTATGAGGTTTGG | | 31 |
| 26d | GTACCCAAACCAATGAGGATCGTCAGTTTGAA | | 32 |
|  | TTCAAACTGACGATCCTCATTGGTTTGG | | 28 |
| 26e | GTACCCAAACCGAACATTTTGATCCGATAATTTAT | | 35 |
|  | ATAAATTATCGGATCAAAATGTTCGGTTTGG | | 31 |
| 27 | GTACCCAAACCGTTCCACCTTATATAACCGGTGGAATTCGGTAT | | 44 |
|  | ATACCGAATTCCACCGGTTATATAAGGTGGAACGGTTTGG | | 40 |
| 28 | GTACCCAAACCGCTTATCATTTCTTTCGATTG | | 32 |
|  | CAATCGAAAGAAATGATAAGCGGTTTGG | | 28 |
| 29 | GTACCCAAACCATGGTGTATTGG | | 29 |
|  | CCAATACACCATGGTTTGG | | 19 |
| 32.1A | GTACCCAAACCGGTCCAGAAACACTTTGGGAACTGGAC | | 38 |
|  | GTCCAGTTCCCAAAGTGTTTCTGGACCGGTTTGG | | 34 |
| 32.1B | GTACCCAAACCGGTCCAGAACCATTATGGGTAGTAGAAACT | | 41 |
|  | AGTTTCTACTACCCATAATGGTTCTGGACCGGTTTGG | | 37 |
| 32.2 | GTACCCAAACCGGTCCAGAATTAATTATTCCATTTATAAGTGGCGGTGTTCCAGCA | | 56 |
|  | TGCTGGAACACCGCCACTTATAAATGGAATAATTAATTCTGGACCGGTTTGG | | 52 |
| 35.1 | GTACCCAAACCTATGGACATTATTCACAACGTTTAGGA | | 38 |
|  | TCCTAAACGTTGTGAATAATGTCCATAGGTTTGG | | 34 |
| 35.2 | GTACCCAAACCTATTATATATCACAAAGACTTGGT | | 35 |
|  | ACCAAGTCTTTGTGATATATAATAGGTTTGG | | 31 |
| 36.1 | GTACCCAAACCTGGTTTCCTATAAAAGAATATCGTGGTGGATTAATGGAAGTT | | 53 |
|  | AACTTCCATTAATCCACCACGATATTCTTTTATAGGAAACCAGGTTTGG | | 49 |
| 36.2a | GTACCCAAACCTGGTATCCTGTGAAAGAATTTCATTATGATGAACCGTTAGAGATT | | 56 |
|  | AATCTCTAACGGTTCATCATAATGAAATTCTTTCACAGGATACCAGGTTTGG | | 52 |
| 36.2b | GTACCCAAACCTGGTTTCCAGTGAAAGAATTCCATTATGATGGACCACTTGAAGTG | | 56 |
|  | CACTTCAAGTGGTCCATCATAATGGAATTCTTTCACTGGAAACCAGGTTTGG | | 52 |
| 36.2c | GTACCCAAACCTGGTCTCCTGTCAAAGAATTTCATTATGATGAACCAATAGAAGTG | | 56 |
|  | CACTTCTATTGGTTCATCATAATGAAATTCTTTGACAGGAGACCAGGTTTGG | | 52 |
| 37 | GTACCCAAACCTGGACTGATTTT | | 23 |
|  | AAAATCAGTCCAGGTTTGG | | 19 |
| 38a | GTACCCAAACCGTTTTAGCTGATTAT | | 26 |
|  | ATAATCAGCTAAAACGGTTTGG | | 22 |
| 38b | GTACCCAAACCCAAGCTATATTAGCTGATTAC | | 32 |
|  | GTAATCAGCTAATATAGCTTGGGTTTGG | | 28 |
| 39 | GTACCCAAACCTTCACTCGTCCATATGGT | | 29 |
|  | ACCATATGGACGAGTGAAGGTTTGG | | 25 |
| 40a | GTACCCAAACCTTTCTGTTAGCTTTACCGTCACCC | | 35 |
|  | GGGTGACGGTAAAGCTAACAGAAAGGTTTGG | | 31 |
| 40b | GTACCCAAACCTTTCTACTTGGTCTACCGCCTAAAGTTGAACAT | | 44 |
|  | ATGTTCAACTTTAGGCGGTAGACCAAGTAGAAAGGTTTGG | | 40 |
| 40c | GTACCCAAACCTTTCTACTTGGTTTACCACCATCACTTAGACAACAT | | 47 |
|  | ATGTTGTCTAAGTGATGGTGGTAAACCAAGTAGAAAGGTTTGG | | 43 |
| 40d | GTACCCAAACCTTCATTTTAGGGCTACCAGCACCAACTAGATTTCATTCG | | 50 |
|  | CGAATGAAATCTAGTTGGTGCTGGTAGCCCTAAAATGAAGGTTTGG | | 46 |
| 41 | GTACCCAAACCTTCTTTTGTAATCCAATGGGATGCGTT | | 38 |
|  | AACGCATCCCATTGGATTACAAAAGAAGGTTTGG | | 34 |
| 42 | GTACCCAAACCCCTTGGACATTACGTGACCCACTGAATTGTTGCTTGGATAATGCTAAATGTTGT | | 65 |
|  | ACAACATTTAGCATTATCCAAGCAACAATTCAGTGGGTCACGTAATGTCCAAGGGGTTTGG | | 61 |
| 43a | GTACCCAAACCGCAAGTTTAGCATATTTT | | 29 |
|  | AAAATATGCTAAACTTGCGGTTTGG | | 25 |
| 43b | GTACCCAAACCGCAAGTTTATCCTATTTT | | 29 |
|  | AAAATAGGATAAACTTGCGGTTTGG | | 25 |
| 47 | GTACCCAAACCGGCAAATTTTTCATGTTAGGA | | 32 |
|  | TCCTAACATGAAAAATTTGCCGGTTTGG | | 28 |
| 48 | CGTTGTGGGTACCCAAACCTATTATACAAATTTGAAAACAATTG | | 44 |
|  | GGTCGTACCAGATCCCCCACCATATCTCATCACATTAGG | | 39 |
