# Supplementary figures and images for: Towards deorphanizing G protein-coupled receptors of Schistosoma mansoni using the MALAR yeast two-hybrid system
Source: Parasitology. 2019 Dec 16;147(8):865–72. doi: 10.1017/S0031182019001756 (PMC7284817; doi:10.1017/S0031182019001756)

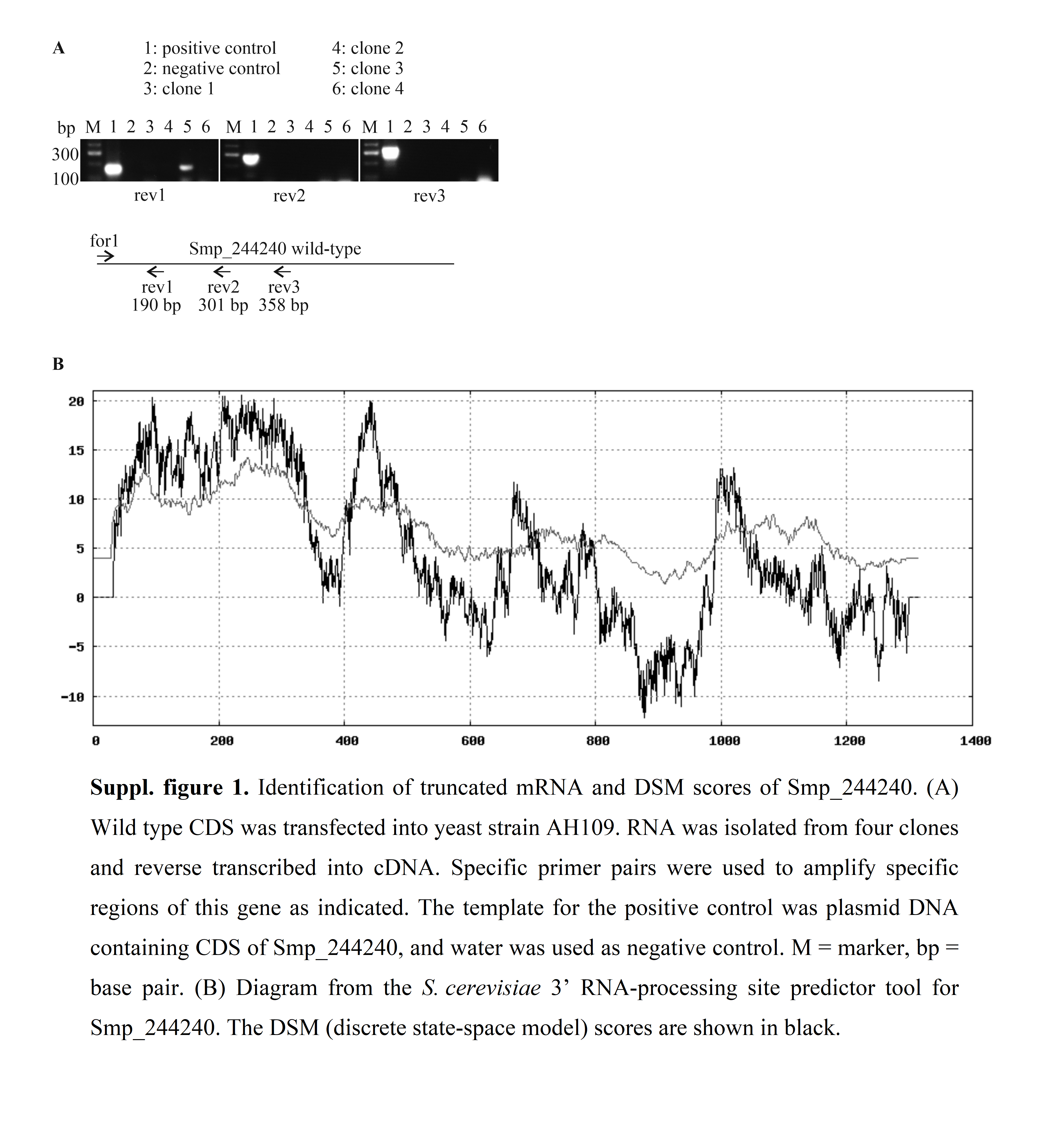

Supplement: Supplementary file 1 [file S0031182019001756sup.zip › S0031182019001756sup003.tif]

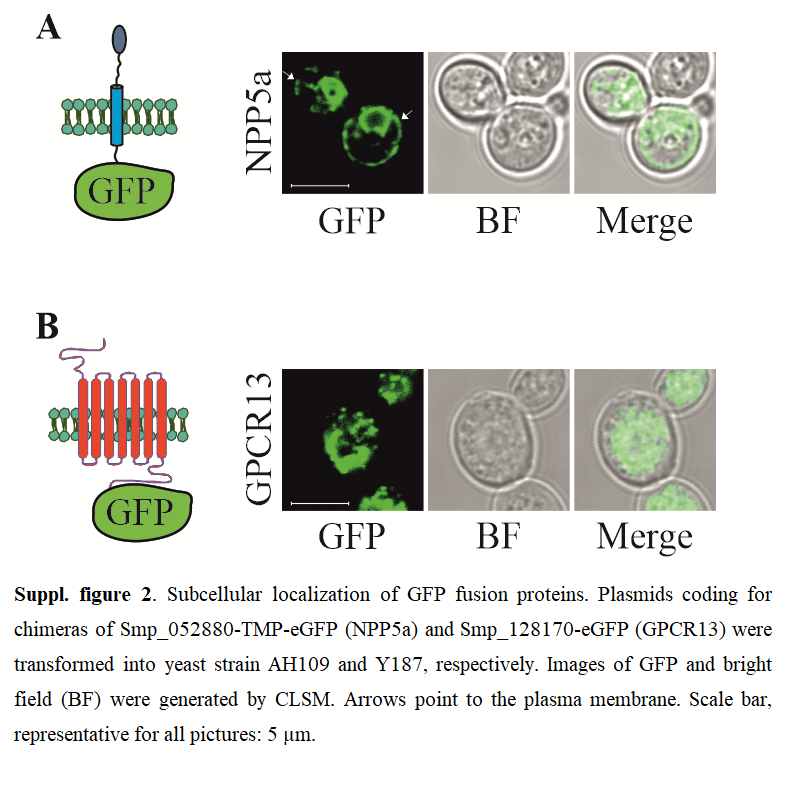

Supplement: Supplementary file 1 [file S0031182019001756sup.zip › S0031182019001756sup005.tif]

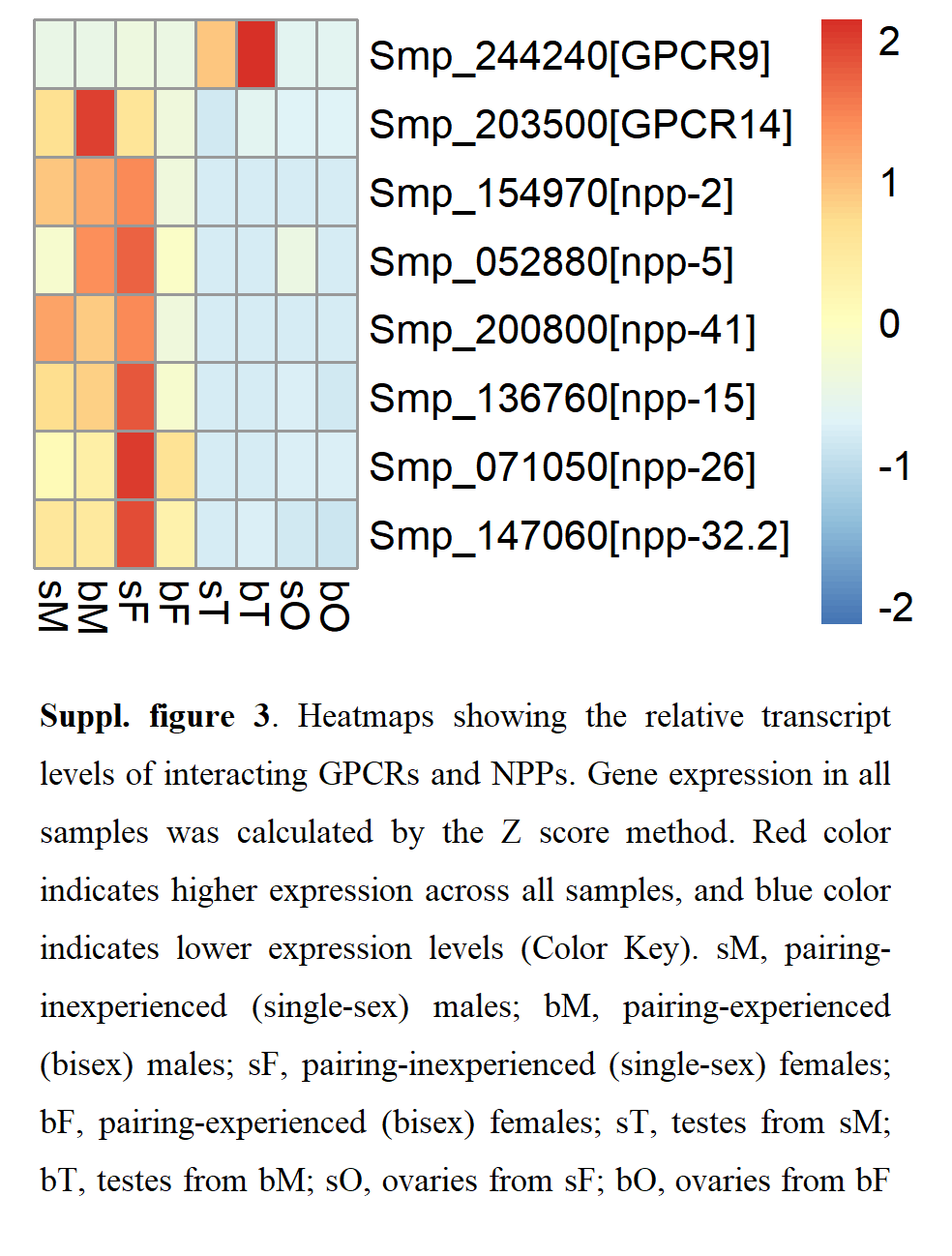

Supplement: Supplementary file 1 [file S0031182019001756sup.zip › S0031182019001756sup006.tif]

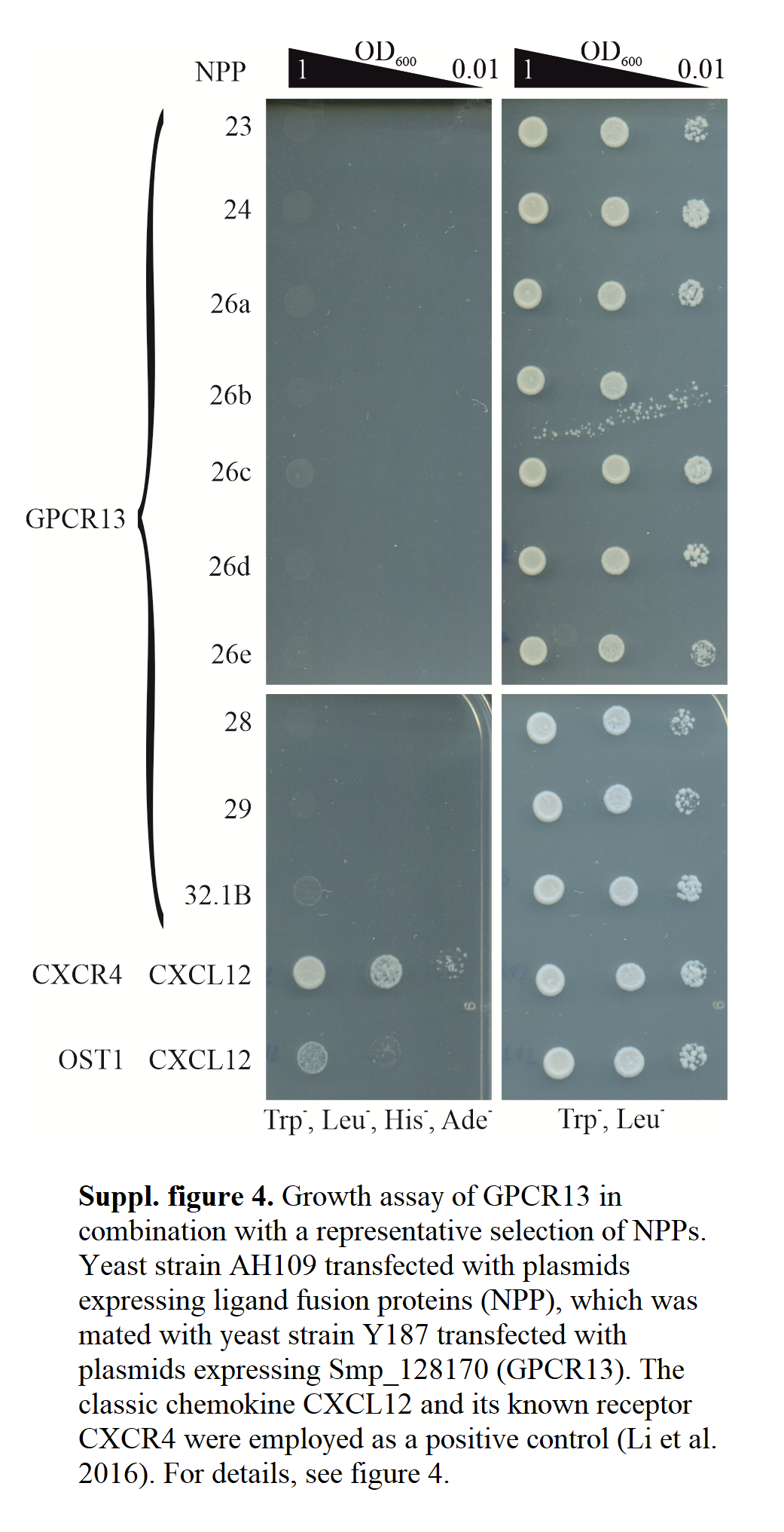

Supplement: Supplementary file 1 [file S0031182019001756sup.zip › S0031182019001756sup007.tif]
